# Focal adhesion molecule Kindlin-1 mediates activation of TGF-β signaling by interacting with TGF-βRI, SARA and Smad3 in colorectal cancer cells

**DOI:** 10.18632/oncotarget.12779

**Published:** 2016-10-20

**Authors:** Jinfeng Kong, Juan Du, Yunling Wang, Mingzi Yang, Jianchao Gao, Xiaofan Wei, Weigang Fang, Jun Zhan, Hongquan Zhang

**Affiliations:** ^1^ Key Laboratory of Carcinogenesis and Translational Research (Ministry of Education), State Key Laboratory of Natural and Biomimetic Drugs, Department of Anatomy, Histology and Embryology, Peking University Health Science Center, Beijing 100191, China; ^2^ Department Pathology, Peking University Health Science Center, Beijing 100191, China

**Keywords:** Kindlin-1, colorectal carcinoma, TGF-β receptor I, Smad3, Smad anchor for receptor activation (SARA)

## Abstract

Kindlin-1, an integrin-interacting protein, has been implicated in TGF-β/Smad3 signaling. However, the molecular mechanism underlying Kindlin-1 regulation of TGF-β/Smad3 signaling remains elusive. Here, we reported that Kindlin-1 is an important mediator of TGF-β/Smad3 signaling by showing that Kindlin-1 physically interacts with TGF-β receptor I (TβRI), Smad anchor for receptor activation (SARA) and Smad3. Kindlin-1 is required for the interaction of Smad3 with TβRI, Smad3 phosphorylation, nuclear translocation, and finally the activation of TGF-β/Smad3 signaling pathway. Functionally, Kindlin-1 promoted colorectal cancer (CRC) cell proliferation in vitro and tumor growth in vivo, and was also required for CRC cell migration and invasion via an epithelial to mesenchymal transition. Kindlin-1 was found to be increased with the CRC progression from stages I to IV. Importantly, raised expression level of Kindlin-1 correlates with poor outcome in CRC patients. Taken together, we demonstrated that Kindlin-1 promotes CRC progression by recruiting SARA and Smad3 to TβRI and thereby activates TGF-β/Smad3 signaling. Thus, Kindlin-1 is a novel regulator of TGF-β/Smad3 signaling and may also be a potential target for CRC therapeutics.

## INTRODUCTION

Colorectal cancer (CRC) is one of the leading cancers worldwide [1]. However, mechanism underlying CRC progression is not completely clear. The progression of CRC is mediated by multiple signaling pathways including the transforming growth factor-β (TGF-β), Wnt, Hedgehog and HOX signalings [2]. Among them, TGF-β and Wnt signalings have been recognized as the most important pathways that regulate CRC progression [2–4]. TGF-β1 was known to initiate its cellular response by activation of TGF-β receptor I (TβRI), which interacted with R-Smads (receptor-phosphorylated SMAD) proteins including Smad2/3, resulting in phosphorylation of Smad2/3. The activated R-Smads form a hetero-trimeric complex with Co-Smad (mainly Smad4), which then translocates to the nucleus to bind to the target DNA and activates the transcription of more than 300 target genes including p21, p53, c-Myc and Snail [2, 5]. However, TGF-β signaling was found to be controlled at various layers. For example, a previous study reported that SARA (Smad anchor for receptor activation) interacts directly with Smad2/3 and functions to recruit Smad2/3 to the TGF-β receptor, suggesting that interaction of TβRI with Smads requires molecules functioned as adaptors or linkers [6]. These sophisticated regulations of TGF-β signaling increased suitability to spatial and temporal control of its target genes.

Kindlin family proteins, composed of Kindlin-1, 2 and 3, are a group of FERM domain-containing adaptor molecules that interact with the cytoplasmic component of integrins and regulate cell-matrix connections. Kindlin-1 is mainly expressed in adult tissues originating from ectoderm/endoderm [7]. Kindlin-1 was known to be essential for maintaining the structure of cell-matrix adhesion, such as focal adhesions and podosomes [8]. Loss of Kindlin-1 or mutations of Kindlin-1 caused Kindler Syndrome characterized by skin blistering, atrophy, and photosensitivity [9–11]. Kindlin-1 was found to be upregulated by TGF-β in HMEC cells [12]. Sin et al reported that Kindlin-1 regulates breast cancer lung metastasis and lung tumorigenesis through regulation of TGF-β signaling [13]. Recently, Kindlin-1was found to control cutaneous epithelial stem cell homeostasis by balancing TGF-β-mediated growth-inhibitory signals and Wnt/β-catenin-mediated growth-promoting signals [14]. These findings strongly suggested that Kindlin-1 is a regulator of TGF-β signaling. However, how Kindlin-1 regulates TGF-β signaling remains unclear.

We here identified that Kindlin-1 directly interacts with the key TGF-β/Smad3 signaling components including TβRI, Smad3 and SARA. These findings uncovered an important role of Kindlin-1 in the control of TGF-β/Smad3 signaling pathway in CRC cells. We analyzed an Oncomine dataset and found that CRC patient specimens express higher level of Kindlin-1 compared to the normal tissues. In support, a recent digital transcript profile analysis showed that Kindlin-1 is a potential novel prognostic marker for CRC patients [15], suggesting that Kindlin-1 may be involved in the progression of CRC. However, the functional role and the molecular mechanism underlying Kindlin-1 regulation of CRC progression remained unknown.

In the present study, we demonstrated that Kindlin-1 promotes tumor growth and is required for TGF-β-induced migration in CRC cells. For clinical relevance, we found that Kindlin-1 protein levels are enhanced with the CRC progression from lower stages to higher ones. Importantly, higher Kindlin-1 level predicted a worse prognosis in CRC patients. Intriguingly, Kindlin-1 was found to form a molecular complex with TβRI, SARA and Smad3 to control the activation of TGF-β/Smad3 signaling pathway.

## RESULTS

### Kindlin-1 expression increases during CRC progression and higher Kindlin-1 expression level is correlated with poor prognosis in CRC patients

**Figure 1:**
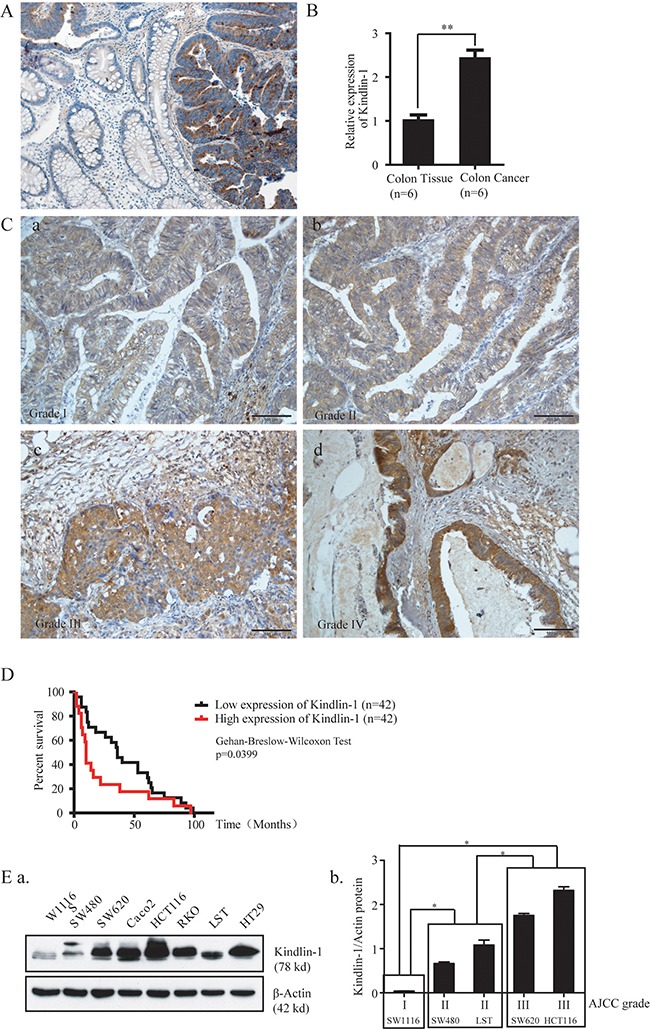
Kindlin-1 expression is correlated with CRC progression and patient outcome

Colorectal benign polyps and adenomas could develop into atypical hyperplasia, in situ carcinoma, and further malignant tumors, constituting a dogma for adenoma-carcinoma transition underlying etiology of colorectal carcinomas (CRC) [16, 17]. To elucidate the possible role of Kindlin-1 in the development of CRC, we examined the expression profile of Kindlin-1 in CRC patient specimens by immunohistochemical (IHC) staining in paired normal mucosa and tumor tissues (Figure 1A and 1B). Kindlin-1 was absent or barely detectable in the normal mucosa crypts and was raised in primary colorectal carcinomas. Interestingly, Kindlin-1 expression was increased with CRC progression from AJCC grades I to IV (Figure 1C). Intriguingly, overexpression of Kindlin-1 in CRC was correlated to a shorter survival in Kaplan-Meier's survival analysis using the Reid colon dataset of Oncomine (Figure 1D), suggesting that Kindlin-1 might be an important prognostic marker for CRC patients. To examine the Kindlin-1 expression profile in CRC cell lines we detected Kindlin-1 protein levels in SW1116, SW480, SW620, Caco2, HCT116, RKO, LST and HT29 cells using Western blot analysis (Figure 1E.a). According to the AJCC grade, SW1116 is classified as AJCC I (low malignancy) and SW480 and LST as AJCC II, SW620 and HCT116 are classified as AJCC III (high malignancy). Thereby Kindlin-1 expression was found increased in CRC cell lines with higher malignancy (Figure 1E.b).

Kindlin-1 expression in colorectal cancer patient specimens was examined by IHC. **A.** Kindlin-1expression level is higher in colon cancer tissue than in normal colon tissue. **B.** Kindlin-1expression levels in normal colon mucosa and tumor tissue from the same pair of patient were quantified (p=0.005). **C.** The expression level of Kindlin-1 in colorectal cancer tissues is correlated with AJCC grade. a. AJCC grade I. b. AJCC grade II. c. AJCC grade III. d. AJCC grade IV. **D.** High expression level of Kindlin-1 predicts a shorter overall survival for patients with colorectal cancer in Reid Colon Dataset of Oncomine (p=0.0399, Gehan-Breslow-Wilcoxon test). **E.** Kindlin-1 protein increases during CRC progression. a. The protein expression profile of Kindlin-1 was examined by Western blot analysis in eight colorectal cancer cell lines using an anti-Kindlin-1 antibody, controlled by actin loading. b. Relative protein band intensities were normalized with each β-actin band and were quantified. According to AJCC grade, the differences among groups I, II and III were analyzed by one-way ANOVA analysis and difference between groups are significantly. * indicates *p*<0.05 by Student's *t* test.

### Kindlin-1 promotes CRC cell growth in vitro and in vivo

**Figure 2:**
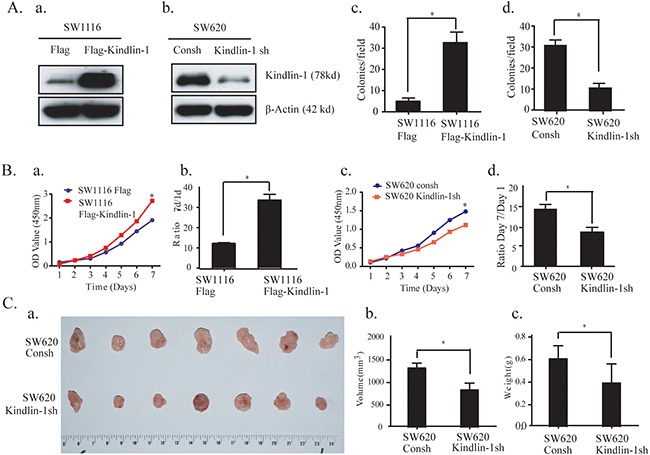
Kindlin-1 promotes CRC cell proliferation in vitro and tumor growth in vivo

To detect the effect of Kindlin-1 on tumor growth, we stably overexpressed Kindlin-1 in SW1116 cells and knocked down Kindlin-1 in SW620 cells (Figure 2A.a and 2A.b). Soft agar colony forming experiment and WST-1 proliferation assay were performed. Results showed that overexpressed Kindlin-1 promoted colony formation of SW1116 cells (Figure 2A.c). In contrast, colony formation capacity was inhibited for SW620 cells when Kindlin-1 was knocked down (Figure 2A.d). Furthermore, overexpressed Kindlin-1 in SW1116 cells promoted cell growth (Figure 2B.a and 2B.b); whereas depletion of Kindlin-1 in SW620 cells decreased cell growth (Figure 2B.c and 2B.d). To examine whether Kindlin-1 is required for tumor growth in vivo, we stably knocked down Kindlin-1 expression in SW620 cells. The control and Kindlin-1 depleted cells were subcutaneously injected into bilateral sides of nude mice. Tumor-bearing mice were euthanized 6 weeks later (Figure 2C. a and b). We found that the volume and weight of the tumors in Kindlin-1 depleted group were lower than that of the control group (*p*<0.05, Figure 2C.c and 2C.d). Taken together, these results indicated that Kindlin-1 is required for CRC cell growth both in vitro and in vivo.

**A.** Kindlin-1 promotes CRC cell anchorage-independent growth. a. Kindlin-1 was overexpressed in SW1116 cells. b. Endogenous Kindlin-1 was depleted by specific small hairpin RNA in SW620 cells. c and d. Soft agar assays were performed using SW1116 with Kindlin-1 overexpression or SW620 with endogenous Kindlin-1 knockdown. Cells were mixed with soft agar and 3 weeks later colonies were photographed. Colonies larger than 50 μm in diameter were counted. Data were shown as mean ± SEM from three independent experiments. * indicates *p*<0.05 by Student's *t* test. **B.** Kindlin-1 promotes CRC cell proliferation. a and b. Cell growth was measured by WST-1 cell viability assay using SW1116 cells with Kindlin-1 overexpression. c and d. Cell growth was measured by WST-1 cell viability assay using SW620 cells with Kindlin-1 knockdown. Data were expressed as mean ± SEM. * indicates *p*<0.05 by Student's *t* test. **C.** Kindlin-1 is required for CRC tumor growth in mice. a. A representative nude mouse in tumor formation experiment. After six weeks, tumors were dissected. The upper panel of tumors was dissected from SW620 control shRNA stable cells, and the lower panel of tumors was dissected from SW620 Kindlin-1 shRNA stable cells. b and c. The average weight and volume of the tumors were quantified. Data were displayed as mean ± SD from a representative experiment. * indicates *p*<0.05 by Student's *t*-test.

### Kindlin-1 promotes epithelial to mesenchymal transition and invasion in CRC cells

**Figure 3:**
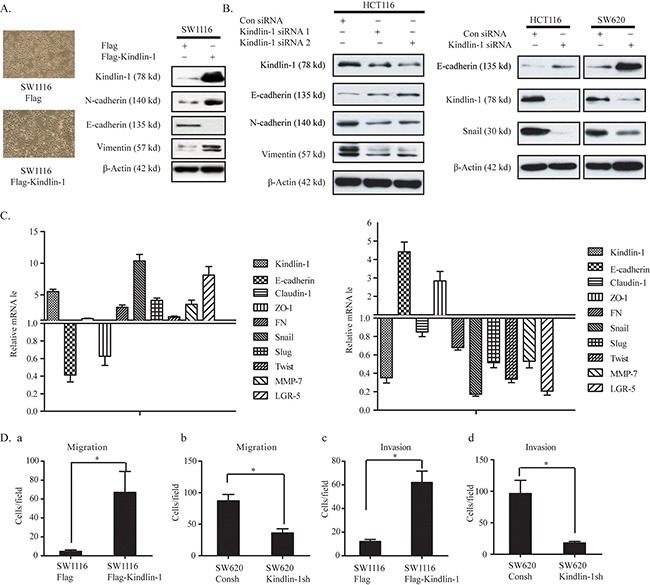
Kindlin-1 promotes CRC cell invasion and migration by stimulating an EMT phenotype

Epithelial-mesenchymal transition (EMT) is essential for the migration and metastasis of epithelial-derived cancer cells to remote organs [18–20]. Kindlin-1 has been shown to be an important adaptor molecule for integrin activation [21]. Loss of Kindlin-1 impairs the cell-cell connections and cell-matrix adhesions, which lead to mucosa epithelial cell partly shedding from the submucosa in neonate intestinal tract, resulting in intestinal disorders [22]. These findings show that Kindlin-1 is necessary for the maintenance of normal intestinal function. However, the role of Kindlin-1 in the regulation of CRC EMT and invasion is still unclear. To this end, we found that the morphology of Kindlin-1 overexpressed SW1116 cells exhibited a mesenchymal-like phenotype (Figure 3A Left panel). Meanwhile, the epithelial cell marker E-cadherin was downregulated while the mesenchymal cell markers N-cadherin and Vimentin were up-regulated (Figure 3A Right panel). Likewise, when we knocked down Kindlin-1 expression in HCT116 and SW620 cells, the epithelial cell marker E-cadherin was restored and EMT regulatory transcriptional factor Snail and mesenchymal markers N-cadherin and Vimentin were downregulated (Figure 3B). In contrast, the mRNA levels of epithelial and mesenchymal markers were also examined using qPCR. Results showed that epithelial markers E-cadherin and ZO-1 were downregulated under Kindlin-1 overexpression, whereas mesenchymal markers including FN, Snail, Slug, Twist, MMP and LGR-5 were upregulated (Figure 3C Left panel). In contrast, depletion of Kindlin-1 in CRC cells restored the expression of epithelial markers E-cadherin and ZO-1, with marked downregulation of mesenchymal markers FN, Snail, Slug, Twist, MMP-7 and LGR-5 (Figure 3C Right panel). Furthermore, the effect of Kindlin-1 on CRC cell migration and invasion was evaluated using Kindlin-1 stably transfected cells in Transwell assays. Results showed that overexpression of Kindlin-1 promoted SW1116 cell migration and invasion capacity (Figure 3D.a and 3D.c). Oppositely, depletion of Kindlin-1 obviously reduced the migration and invasion capacity of SW620 cells (Figure 3D.b and 3D.d). These data indicated that Kindlin-1 is an important controller for CRC cell migration and invasion that constituting the metastatic capability of colorectal cancer.

**A.** Left panel: Morphological changes for CRC cells with or without Kindlin-1 expression were observed by a phase contrast microscope. Right panel: SW1116 cells were transfected with Flag or Flag-Kindlin-1 for 48 h. Cell lysates were subjected to Western blot analysis using antibodies as indicated. **B.** Depletion of Kindlin-1 increases epithelial markers and reduces mesenchymal markers in CRC cells. Kindlin-1 siRNA1 and 2 (left panel) or Kindlin-1 siRNA (1+ 2) (right panel) were transfected into HCT116 or SW620 cells, and cell lysates were subjected to Western blot analysis using indicated antibodies. **C.** The levels of EMT markers were detected by qPCR in Kindlin-1 overexpressed (Left panel) or knocked down (right panel) in HCT 116 cells. **D.** Migration and invasion assays were performed in SW1116 Flag and SW1116 Flag-Kindlin-1 cells. Kindlin-1 is required for CRC cell migration and invasion. Migration and invasion assays were performed using SW620 cells with control shRNA or SW620 Kindlin-1 shRNA. a and c. Gain-of-function experiments. b and d. Loss-of-function experiments. Data were displayed as mean ± SEM from three independent experiments. * indicates *p*<0.05 by Student's *t* test.

### Kindlin-1 interacts with TβRI and Smad3 in CRC cells

**Figure 4:**
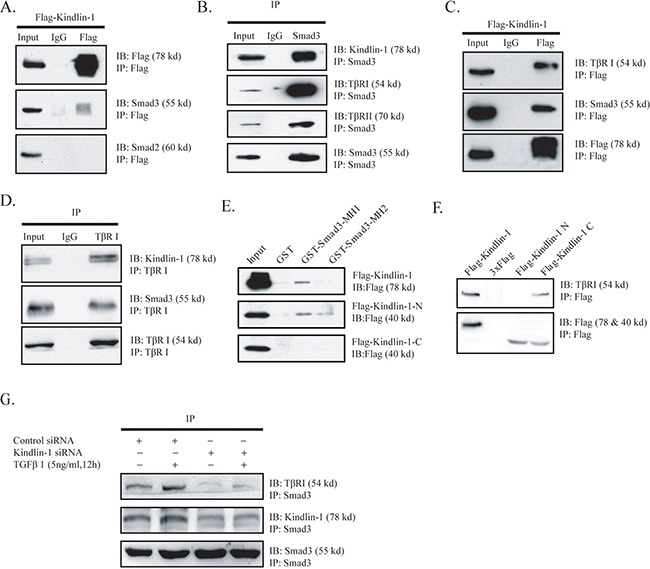
Kindlin-1 is required for the interaction of TβRI with Smad3 in CRC cells

To elucidate the role of Kindlin-1 in TGF-β signaling during CRC progression, we examined whether Kindlin-1 may interact with Smad proteins using co-immunoprecipitation (co-IP) in HCT116 cells transiently transfected with Flag-Kindlin-1. Interestingly, we found that exogenous Flag-Kindlin-1 interacted with endogenous Smad3 but not with Smad2 in a co-IP assay using Flag antibody (Figure 4A). We then examined whether Kindlin-1 complexes with TβRI and Smad3. To this end, we identified that Flag-Kindlin-1 is able to co-immunoprecipitate endogenous Smad3 and TβRI in HCT116 cells (Figure 4B). Furthermore, we did reciprocal experiments using anti-Smad3 and anti-TβRI antibodies separately. Anti-Smad3 could co-immunoprecipitate endogenous Kindlin-1 and TβRI in HCT116 cells (Figure 4C). Likewise, anti-TβRI could co-immunoprecipitate endogenous Kindlin-1 and Smad3 in HCT116 cells (Figure 4D). These results clearly indicated that there is a Kindlin-1 -Smad3-TβRI molecular complex in CRC cells.

**A.** Kindlin-1 is associated with Smad3 but not Smad2. HCT116 cells were transfected with Flag-Kindlin-1 for 48 h, then proteins were extracted for co-IP assays using an anti-Flag antibody, followed by Western blot analysis using indicated antibodies. **B.** Smad3 is associated with Kindlin-1, TβRI and TβRII. HCT116 cell lysates were extracted and co-IP was performed using a Smad3 antibody, followed by Western blot analysis using indicated antibodies. **C.** Kindlin-1 is associated with Smad3 and TβRI. HCT116 cells were transfected with Flag-Kindlin-1 for 48 h, then proteins were extracted for co-IP assays using an anti-Flag antibody, followed by Western blot analysis using indicated antibodies. **D.** TβRI is associated with Kindlin-1 and Smad3. Protein was extracted from HCT116 cells and co-IP assays were performed using anti-TβRI antibody, followed by Western blot analysis using indicated antibodies. **E.** Smad3-MH1 domain binds to Kindlin-1 N-terminal domain. GST, GST-Smad3-MH1 and GST-Smad3-MH2 were expressed and purified from *E.coli*. The full-length, N-terminal and C-terminal domains of Kindlin-1 were expressed in HCT116 cells separately. GST pull-down assays were performed, followed by Western blot analysis using an anti-Flag antibody. **F.** Kindlin-1 C-terminal domain binds to TβRI. Flag, Flag-Kindlin-1-N and Flag-Kindlin-1-C expression vectors were transfected into HCT116 cells for 48h, and then co-IP assays were performed using an anti-Flag antibody, followed by Western blot analysis using indicated antibodies. **G.** Depletion of Kindlin-1 jeopardizes TβRI association with Smad3 in CRC cells. Control or Kindlin-1 siRNA was transfected into HCT116 cells for 36h followed by treatment of TGF-β1 (5ng/ml) for another 12h. Proteins were extracted for co-IP assays using an anti-Smad3 antibody, followed by Western blot analysis using indicated antibodies.

To determine the interacting regions between Kindlin-1 and Smad3, we constructed Flag tagged N-terminal (aa 1 to 350) and C-terminal (aa 351 to 677) domains of Kindlin-1 with Flag tag, named Flag-Kindlin-1-N and Flag-Kindlin-1-C. We also made different GST fused Smad3 segments, named GST-Smad3-MH1 (aa 1 to 200) and GST-Smad3-MH2 (aa 201 to 425). The truncation constructs of Kindlin-1 were transiently transfected into SW1116 cells and cell lysates were pulled down using different GST-fused Smad3 fragments. Results showed that the N-terminal domain of Kindlin-1 interacted with the MH1 (MAD-homology 1) domain of Smad3 (Figure 4E). Furthermore, we transfected HCT116 cells with different Kindlin-1 truncated constructs and performed a co-IP assay using an anti-Flag antibody. Result showed that the C-terminal domain of Kindlin-1 had an interaction with TβRI (Figure 4F). These data indicated that Kindlin-1 might act as an adaptor molecule between Smad3 and TβRI. Taken together, these findings suggested that Kindlin-1 may play an important role in recruiting Smad3 to TβRI and finally Smad3-Kindlin-1-TβRI complex leads to Smad3 phosphorylation.

To support the notion that Kindlin-1 recruits Smad3 to TβRI, we knocked down Kindlin-1 by siRNA in HCT116 cells to examine the interaction between Smad3 and TβRI. Intriguingly, we found that the interaction between Smad3 and TβRI was weakened under the depletion of Kindlin-1 (Figure 4F), which could be partially recovered by TGF-β1 stimulation (Figure 4F). Collectively, these results indicated that Kindlin-1 is required for TβRI interaction with Smad3 by forming a Smad3-Kindlin-1 -TβRI triple molecular complex.

### Kindlin-1 mediates TGF-β/Smad3 signaling by activation of Smad3

**Figure 5:**
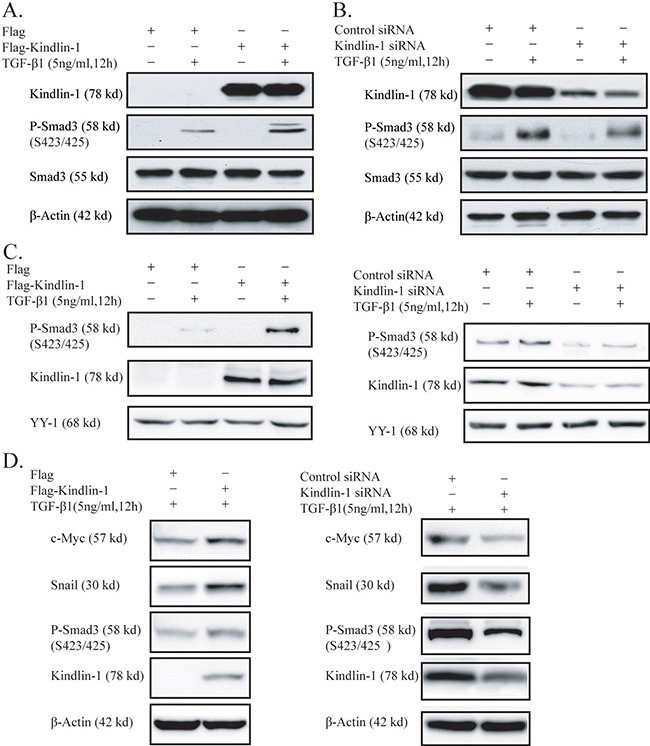
Kindlin-1 activates TGF-β/Smad3 signaling in CRC cells

Smad3 can be activated by phosphorylation at S423/425 sites, then phosphorylated Smad3 translocates into the nucleus and further activates the transcription of target genes [23]. To examine whether Kindlin-1 may play a role in Smad3 activation, we treated SW1116 cells by TGF-β1 or not after Kindlin-1 knockdown. Interestingly, we found that TGF-β1-induced Smad3 phosphorylation could be further enhanced by overexpression of Kindlin-1 (Figure 5A). However, overexpression of Kindlin-1 alone could not lead to Smad3 phosphorylation without addition of TGF-β1, indicating that Kindlin-1 is able to activate Smad3 only upon the presence of TGF-β1. In contrast, when we knocked down Kindlin-1 in HCT116 cells, the phosphorylated Smad3 was remarkably reduced even with the presence of TGF-β1 (Figure 5B), pointing out that Kindlin-1 is required for TGF-β1-stimulated Smad3 activation. To further investigate the effect of Kindlin-1 on the nuclear translocation of phosphorylated Smad3, we extracted the nuclear proteins from SW1116 cells with Kindlin-1 expression. Importantly, we found that the nuclear translocation of phosphorylated Smad3 induced by TGF-β1 could be maintained only under Kindlin-1 overexpression (Figure 5C Left panel), suggesting that Kindlin-1 is required for the nuclear translocation of phosphorylated Smad3. In addition, depletion of Kindlin-1 could not maintain Smad3 activation in the nuclei even with the presence of TGF-β1 (Figure 5C Right panel). We then continued to examine whether Kindlin-1 could upregulate TGF-β/Smad3 signaling target genes in CRC cells. We transfected Flag-Kindlin-1 expression vector into SW1116 cells and Western blot analysis demonstrated that Smad3 was activated and the TGF-β/Smad3 signaling target genes c-myc and Snail were upregulated (Figure 5D Left panel). In contrast, depletion of Kindlin-1 by siRNA led to reduced activation of Smad3 and downregulated c-myc and Snail expression in HCT116 cells (Figure 5D Right panel). These findings clearly indicated that Kindlin-1 could mediate the activation of TGF-β/Smad3 signaling pathway and upregulate its target genes in CRC cells.

**A.** Kindlin-1 potentiates TGF-β1 activation on Smad3. Flag or Flag-Kindlin-1 expression vector was transfected into SW1116 cells for 36h followed by treatment of TGF-β1 (5ng/ml) for another 12h. Proteins were extracted for Western blot analysis using indicated antibodies. **B.** Kindlin-1 is required for TGF-β1 activation on Smad3. Control or Kindlin-1 siRNA was transfected into HCT116 cells for 36h followed by treatment of TGF-β1 (5ng/ml) for another 12h. Proteins were extracted for Western blot analysis using indicated antibodies. **C.** Kindlin-1 is required for translocation of phosphorylated Smad3 into the nucleus. Left panel: Flag or Flag-Kindlin-1 expression vector was transfected into SW1116 cells for 36h followed by treatment of TGF-β1 (5ng/ml) for another 12h. Right panel: Control or Kindlin-1 siRNA was transfected into HCT116 cells for 36h followed by treatment of TGF-β1 (5ng/ml) for another 12h. Nuclear proteins were extracted for Western blot analysis. The presence of YY1 indicates that the fraction was derived from the nuclei. **D.** Kindlin-1 is required for TGF-β1 target gene expression. Left panel: Flag or Flag-Kindlin-1 expression vector was transfected into SW1116 cells for 36h followed by treatment of TGF-β1 (5ng/ml) for another 12h. Western blot analysis was performed using indicated antibodies. Right panel: Control or Kindlin-1 siRNAs was transfected into HCT116 cells, followed by treatment of TGF-β1 (5ng/ml) for another 12h. Western blot analysis was performed using indicated antibodies.

### Kindlin-1 and SARA could compensate to each other in the activation of Smad3 in CRC cells

**Figure 6:**
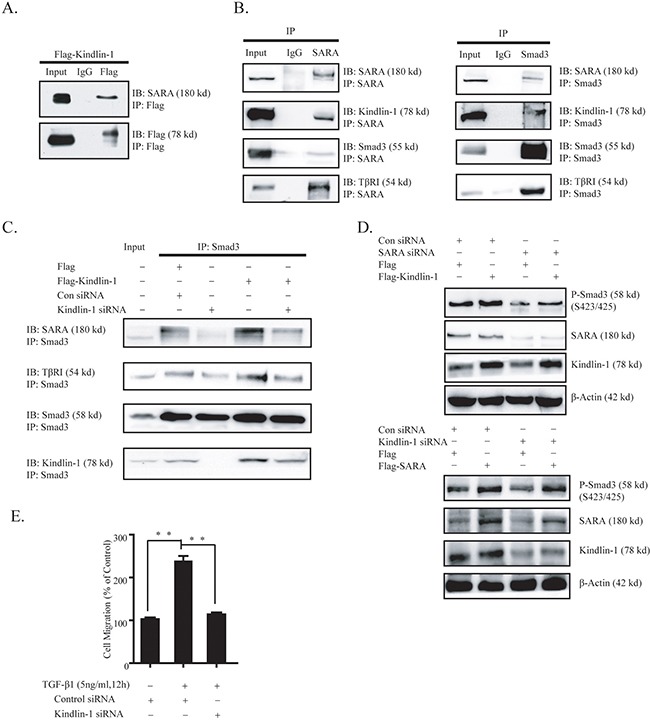
Kindlin-1 forms a complex with SARA, TβRI and Smad3 to activate TGF-β/Smad3 signaling in CRC cells

Smads activity in TGF-β signaling is modulated by a number of adaptor proteins including ELF, SARA, Filamin and microtubules [2]. Among these adaptors, SMAD anchor for receptor activation (SARA) was originally identified as a protein that recruits non-phosphorylated Smad2/3 to the activated receptors (TβRI and TβRII), followed by phosphorylation and activation of Smad2/3 that lead to cell growth, differentiation and cell fate specification [24, 25]. In our study, we found that Kindlin-1 played a similar role as SARA to recruit Smad3 to TβRI for phosphorylation. To identify the relationship between Kindlin-1 and SARA, we did a co-IP assay with an anti-Flag antibody using lysates from HCT116 Flag-Kindlin-1 cells followed by immunoblotting with an anti-SARA antibody. Interestingly, we found an interaction between Kindlin-1 and SARA (Figure 6A). Given that we showed that Kindlin-1 formed a triple molecular complex with Smad3 and TβRI (Figure 4), we thus wondered whether SARA may complex with the above three proteins. Intriguingly, we successfully co-immunoprecipitated Kindlin-1, Smad3 and TβRI with an anti-SARA antibody (Figure 6B Left panel), and co-immunoprecipitated SARA, Kindlin-1 and TβRI with an anti-Smad3 antibody (Figure 6B Right panel). These data suggested that Kindlin-1, SARA, Smad3 and TβRI may form a molecular complex underneath cell membrane to mediate TGF-β/Smad3 signaling.

**A.** Kindlin-1 interacts with SARA in CRC cells. HCT116 cells were transfected with Flag or Flag-Kindlin-1 expression vector for 48 h, later proteins were extracted for co-IP assays using an anti-Flag antibody, followed by Western blot analysis using indicated antibodies. **B.** Association of SARA, Kindlin-1, Smad3 and TβRI in living CRC cells. Proteins were extracted from HCT116 cells and co-IP assays were performed using anti-TβRI (Left panel) or anti-Smad3 (Right panel) antibodies, followed by Western blot analysis using indicated antibodies. **C.** Kindlin-1 regulates interaction between Kindlin-1 and TβRI in CRC cells. HCT116 cell lysates with Kindlin-1 overexpression or Kindlin-1 knockdown were co-immunoprecipitated using an anti-Smad3 antibody, controlled by empty vector or control siRNA. Western blot assays were performed using indicated antibodies. **D.** Upper panel: Kindlin-1 rescues SARA-depletion caused Smad3 inactivation. Control or SARA siRNA was transfected into HCT116 cells with or without Kindlin-1 overexpression. Western blot assay was performed using indicated antibodies. Lower panel: SARA rescues Kindlin-1-depletion caused Smad3 inactivation. Control or Kindlin-1 siRNA was transfected into HCT116 cells with or without SARA overexpression. Western blot assay was performed using indicated antibodies. **E.** Kindlin-1 is required for TGF-β-induced CRC cell migration. Control or Kindlin-1 siRNA was transfected into HCT116 cells. Twenty-four hours later cells were treated with TGF-β1 for another 12 h, then cell migration assay was performed. Data were expressed as mean ± SEM from three independent experiments, *p*<0.01.

To scrutinize the effect of Kindlin-1 on the molecular interactions of Smad3 with SARA and TβRI, we examined associations among Smad3, SARA and TβRI in HCT116 cells with depletion or overexpression of Kindlin-1. We found that less SARA and TβRI can be co-immunoprecipated by a Smad3 antibody under Kindlin-1 depletion (Figure 6C). Whereas more SARA and TβRI can be co-immunoprecipated by a Smad3 antibody under Kindlin-1 overexpression (Figure 6C). Interestingly, less SARA and TβRI co-immunoprecipated due to the depletion of Kindlin-1 could be partially reversed by re-expression of Kindlin-1 (Figure 6C). These results suggested that Kindlin-1 is required for the molecular interactions among Smad3, SARA and TβRI.

Given that Kindlin-1 and SARA are both adaptor molecules that mediate the activation of Smad3 in TGF-β/Smad3 signaling, we wondered whether these two molecules could replace each other in activation of Smad3 in CRC cells. To this end, we knocked down SARA in HCT116 cells and found that P-Smad3 (S423/425) was reduced in HCT116 cells, and the decreased Smad3 activation can be rescued by Kindlin-1 overexpression (Figure 6D Upper panel). Likewise, depletion of Kindlin-1 reduced P-Smad3 (S423/425) and this effect could be rescued by SARA overexpression (Figure 6D Lower panel). These results indicated that Kindlin-1 and SARA could support and compensate to each other in the activation of Smad3.

## DISCUSSION

**Figure 7:**
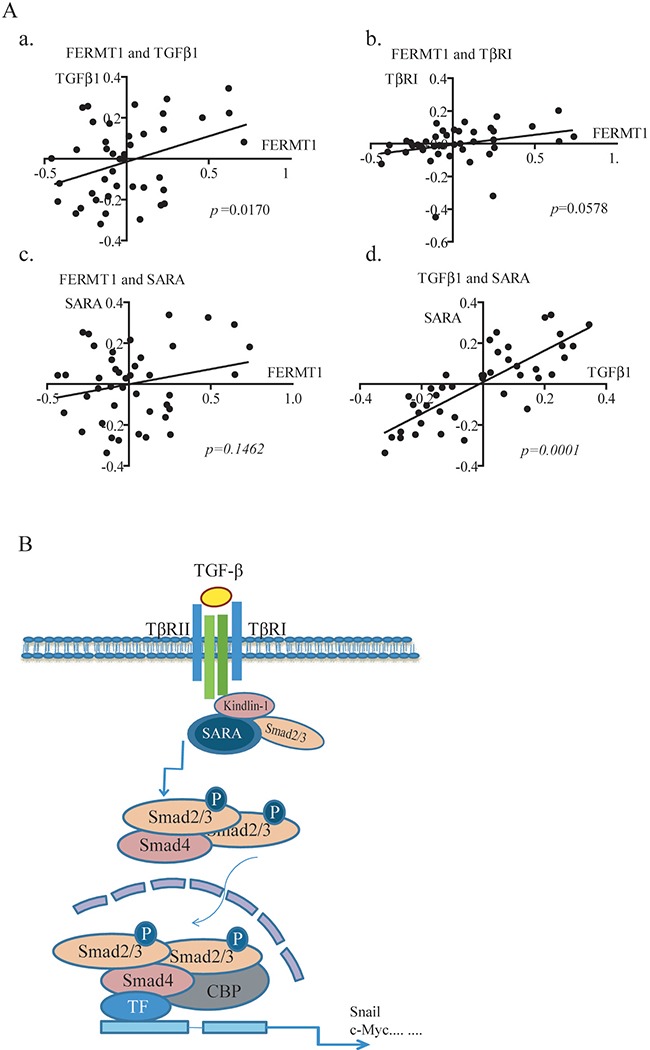
Kindlin-1 is required for TGF-β/Smad3 signaling in CRC progression - A working model

In this study, we reported an unidentified mechanism accounting for Kindlin-1 regulation of CRC progression by demonstrating that Kindlin-1 is a new component and regulator of TGF-β/Smad3 signaling (Figure 7). TGF-β/Smad3 signaling was known to be one of the most important signaling pathways in the control of colorectal cancer progression [23]. For important clinical relevance higher Kindlin-1 expression level was found to correspond to a worse prognosis in CRC patients. Interestingly, Kindlin-1 was known to be regulated by TGF-β1 [12] and reversely Kindlin-1 is a key player in the activation of TGF-β/Smad3 signaling as demonstrated in this report. Phosphorylation of the R-Smads is the committed step in activation of TGF-β/Smad3 signaling. Recruitment of R-Smads to the receptor TβRI is regulated by a number of adaptor proteins such as SARA, Filamin, Microtubules and ELF [24–26]. SARA activates TGF-β/Smad3 signaling by recruiting non-phosphorylated Smad2/3 to the activated receptors for phosphorylation. ELF deficiency results in mislocalization of Smad3, Smad4, and consequent loss of TGF-β/Smad3 signaling activity [25]. Intriguingly, we identified in this investigation that integrin-interacting focal adhesion molecule Kindlin-1 is a new adaptor molecule that integrates TβRI, SARA and Smad3 together, thereby regulating activation of TGF-β/Smad3 signaling (Figures 4–6). On one hand, Kindlin-1 interacts with TβRI through its C-terminal domain, at the same time the N-terminal domain of Kindlin-1 interacts with the MH1 domain of Smad3, suggesting that Kindlin-1 functions as a molecular linker recruiting Smad3 to TβRI. On the other hand, Kindlin-1 promotes Smad3 phosphorylation and the following nuclear translocation, leading to the activation of downstream target genes such as Snail and c-myc.

**A.** Inter-relationship between FERMT1 RNA expression with members of TGF-β1 signaling pathway in Reid Colon Dataset of Oncomine (*p*=0.017). a. FERMT1RNA is correlated with TGF-β1. b. FERMT1RNA showed a tendency with TβRI. c. FERMT1RNA is not correlated with SARA, d. Correlation of TGF-β1 RNA is strongly correlated with SARA. **B.** A working model depicting the relationship between Kindlin-1 and TGF-β/Smad3 signaling pathway. In this model, Kindlin-1interacts with key TGF-β/Smad3 signaling components including TβRI, SARA and Smad3, and functionally as a signaling hub that brings TβRI, SARA and Smad3 together, and is required for active TβRI phosphorylation on Smad3. Without the presence of Kindlin-1, TGF-β/Smad3 signaling cannot be fully activated. Taken together, Kindlin-1 promotes CRC progression through activation of the TGF-β/Smad3 signaling pathway.

Surprisingly, we found that Kindlin-1 is also able to interact with SARA, another molecular adaptor that links Smad3 to TβRI to control the activation of TGF-β/Smad3 signaling. It is tempting to ask why Kindlin-1 and SARA are both involved in TGF-β/Smad3 signaling. To this end, our results showed that Kindlin-1 and SARA could act complementarily in regulating the phosphorylation of Smad3 in CRC cells (Figure 6D). These findings suggested that adaptors may function cooperatively to control the activation of TGF-β/Smad3 signaling. Therefore, more investigations are required in the future to elucidate the individual role of adaptor molecules in mediating TGF-β/Smad3 signaling.

Kindlin-1 was identified in this report to regulate epithelial to mesenchymal transition (EMT) in CRC cells and plays a critical role in the maintenance of epithelial cell phenotype. Enhanced expression of Kindlin-1 promoted EMT-related transcriptional factors Snail, Slug and Twist, which downregulate the epithelial markers including E-cadherin and ZO-1 (Figure 3C left). Intriguingly, Kindlin-1 promoted the expression of LGR-5, a colon cancer stem cell marker [27]. LGR-5 was known to be correlated with CRC lymph node metastasis [28]. Therefore, these findings supported a notion that Kindlin-1 is an important regulator for CRC progression, and modulation of Kindlin-1 may provide a new therapeutic approach to TGF-β-mediated EMT and CRC cell invasion.

It has been reported that Kindlin-2 physically interacts with both TβRI and Smad3, promoting the activation of TGF-β/Smad3 signaling and contributing to the pathogenesis of tubulointerstitial fibrosis [29]. These findings indicated that both Kindlin-1 and Kindlin-2 interact with TβRI and Smad3 and activate TGF-β signaling pathway. It has also been reported that Kindlin-1 is mainly expressed in ectoderm- and endoderm-derived organs [7]. In contrast, Kindlin-2 is mainly located at mesoderm-derived organs [30]. Therefore, we hypothesize that Kindlin-1 and Kindlin-2 may function similarly in organs like colon and kidney. However, opposite roles have been identified for Kindlin-1 and Kindlin-2 in the regulation of lung cancer progression [31]. Importantly, Kindlin-1 has been identified to interact with SARA in the present report, possible interaction of Kindlin-2 with SARA warrants future investigations.

Taking together, we found that Kindlin-1 promotes the CRC growth and invasion by activating TGF-β/Smad3 signaling pathway. Kindlin-1 forms a molecular complex with TβRI, SARA, and Smad3, in which Kindlin-1 in cooperation with SARA acts as a molecular hub linking TβRI and Smad3 together.

## MATERIALS AND METHODS

### Clinical specimens

The Ethics Committee of Peking University Health Science Center has approved for mouse experiments (Permit Number: LA2011-73), and for using human colorectal adenocarcinoma patient tumor tissues (Permit Number: ZRLW-5) in the present study. The procedures for handling mice and human tumor specimens were in accordance with the ethical standard of the Helsinki Declaration of 1975, and the revised version in 1983. We also referred procedures from Workman et al (Workman P, Aboagye EO, Balkwill F, Balmain A, Bruder G, Chaplin DJ, et al. Guidelines for the welfare and use of animals in cancer research. British journal of cancer. 2010;102:1555-77).

Tumor tissue sections (n=63) were obtained from colorectal adenocarcinoma patients who underwent surgery at Peking University Health Science Center between July 2006 and September 2007. Normal colon tissue samples (n=63) were obtained from the same colon cancer patient cohort, which were at least 2 cm apart from the tumors. All patients included in this study were not treated with neoadjuvant and adjuvant therapies.

### Antibodies

These antibodies were used in the present investigation: Kindlin-1 (Millipore, MA), c-myc (Snail (Millipore), E-cadherin (Abcam), N-cadherin (Epitomics), p-Smad3 (Abcam), Smad2 (Epitomics), Smad3 (Epitomics), SARA (Epitomics), TβRI (Santa Cruz, CA), TβRII (Santa Cruz, CA), Vimentin (Epitomics), YY-1 (Santa Cruz, CA), Actin (Santa Cruz), or Flag (Sigma-Aldrich, St. Louis, MO).

### Co-immunoprecipitation assay

Co-immunoprecipitation (co-IP) was performed according to the method described previously [29]. SW1116 or HCT116 cells were transiently transfected with or without expression constructs using Lipofectamine 2000 (Invitrogen, USA). Lysates were prepared in NP40 buffer (Ding Guo, China) with addition of protease inhibitor cocktail (Roche Diagnostics, GmbH), followed by 13,000 rpm centrifugation for 15 min at 4°C to remove cell debris. Protein complexes were obtained by incubation of 500 μg precleared lysates with either 4 μg specific antibodies or normal IgG overnight at 4°C, respectively. Immunoblotting was performed using indicated antibodies as the primary antibodies and Clean-Blot IP detection reagent (HRP, Thermo) as the secondary antibody. The membranes were detected by ECL, as mentioned above.

### Cell proliferation assay

Cell proliferation was determined using WST-1 cell proliferation and cytotoxicity assay kit (Beyotime, Haimen, China). Assays were performed according to the recommended protocol from the supplier.

### Soft agar colony formation assay

The 0.7% soft agar (Sigma) was made and put in 6-well plates as a base layer and the 0.35% soft agar was placed on top mixed with suspended cells (1.0×10^3^ cells per well). Each layer consisted of 3 ml soft agar containing complete growth medium. The cells were cultured for 14 days until formed colonies were visible. The visible colonies were then counted. Each experiment was performed on three replicate samples and repeated for three times.

### In vivo xenograft tumor growth

Balb/c nude mice were implanted subcutaneously into the flank with 1×10^6^ SW620 cells stably expressing Kindlin-1 shRNA and the control vector separately. Tumors were observed every 3 days and continued to record for the indicated time. Tumors were dissected and weighted at day 28 when they reached to approximately 1 cm in diameter.

### Cell migration and invasion assays

Cell migration assay was performed using Transwell chambers (Costar Inc., NY) with 8.0 μm pore size. SW1116 cells stably expressing Flag-Kindlin-1 controlled with empty vector were measured for cell migration towards collagen type I (Sigma). SW620 stably expressing Kindlin-1 shRNA controlled with Con shRNA were measured for cell migration towards collagen type I. Cells were allowed for migration for 9h in migration buffer (RPMI1640, 2 mM CaCl_2_, 1 mM MgCl_2_, 0.2 mM MnCl_2_, and 0.5% BSA) at 37°C in humidified 5% CO_2_. The invasion assay was performed by using the same Transwell chambers with addition of Matrigel (BD Biosciences, Billerica) to the upper surface of the Transwell before adding cells and incubated for 12 h. The Transwell membranes were fixed with 4% formaldehyde for 15 min and stained by crystal violet for 10 min. At last, 6 microscopic fields were randomly chosen for counting the migrated or invaded cells.

### Cell culture and the establishment of Kindlin-1 stably expressing or depletion clones

Colorectal cancer cell lines SW1116, SW620, SW480, Calo2, HCT116, RKO, LST, and HT29 were purchased from ATCC in the USA or Cell Collection Center of Peking Union Medical School. Cells were cultured in RPMI1640 medium (Invitrogen) with 10% FBS and 50 μg/ml gentamycin in 75 cm^2^ culture flasks or 60-mm dishes at 37°C in humidity with 5% (v/v) CO_2_. For establishment of Kindlin-1 stably expressing clones, SW1116 cells were transfected with Flag-Kindlin-1, empty vector. For establishment of Kindlin-1 stably depletion clones, SW620 were transfected with Kindlin-1 shRNA (Forward: TGCCTGAGTGCAGATTGCAATTCAAGAGATTGCA ATCTGCACTCAGGCTTTTTTC, Reverse: TCGAGAAAAAAGCCTGAGTGCA GATTGCAATCTCTTGAATTGCAATCTGCACTCAGGCA) and control shRNA using Lipofectamine. Twenty-four hours after transfection cells were passaged and G418 was added at final concentration of 800 μg/ml. Pooled cells expressing Kindlin-1 were used for functional studies as indicated in the text.

### siRNA inhibition of Kindlin-1 expression

The sequences of the Kindlin-1 siRNA were as follows:

Kindlin-1-siRNA1 : 5′-CAGCUGCUCUUACGAUUUATT-3′

Kindlin-1-siRNA2: 5′-GCCUGAGUGCAGAUUGCAATT-3′ (Shanghai GenePharma Co., Ltd., Shanghai, China). SW620 cells (1.5×10^5^ per well) were seeded in 6-well plates and incubated for 20 h at 37°C. HiperFect Transfection Reagent (Qiagen GmbH, Wetzlar, Germany) was used for siRNA transfection according to the recommended protocol from the supplier. Kindlin-1-siRNA 1 and 2 were both examined and found to be equally well for knocking down the endogenous Kindlin-1. Thus, we applied mixture of Kindlin-1-siRNA 1 and 2 (Ratio 1:1) in the Kindlin-1 depletion experiments.

### Quantitative PCR

Quantitative PCR assays were performed to detect the expression of Kindlin-1, E-cadherin, Claudin-1, ZO-1, Fibronectin, Snail, Slug, Twist, MMP-7 and LGR-5. In brief, total cellular mRNAs were isolated by Trizol (Invitrogen), and 2 μg of total RNA was reverse-transcribed using M-MLV reverse transcriptase (Promega, Madison, WI, USA). Then PCR was performed using Taq PCR MasterMix (TIANGEN, Beijing, China) with the settings as: 94°C 2 min; 94°C 30 s, 60°C 30 s, 72°C 30 s, for 30 cycles; 72°C 5 min. The primers for qPCR were as follows: for human Kindlin-1, forward: 5′-TCATGTTGGAGGAGTGATGC-3′, reverse: 5′-AAG CCAGCA ATGCTTCTGTT-3′; for human E-cadherin, forward: 5′-CTGGGCTGGACCGAG AGA-3′, reverse: 5′-GAAGGTCAGCAGCTTGAACCA-3′; for human Claudin-1, forward: 5′-TCTGGCTATTTTAGTTGCCA CAG-3′, reverse: 5′-AGAGAGCCTG ACCAAATTC GT-3′; for human Fibronectin, forward: 5′-GGTGACA CTTATGAGC GTCCTAAA-3′, reverse: 5′-AACATGTAA CCACCAGTCTCATGTG-3′; for human Snail, forward: 5′-TCGGAAGCCTAACTACAGCGA-3′, reverse: 5′-AG ATGAG CATTGGCAGCGAG-3′; for human Slug, forward: 5′-CGAACTGGACACAC ATACAGTG-3′, reverse: 5′-CTGAGGATCTCTGGTTGTGGT-3′; for human Twist, forward: 5′-GTCCGCAGTCTTACGAG GAG-3′, reverse: 5′-GCTTGAGGGTCTG AATCTTG CT-3′; for human MMP-7, forward: 5′-GTCTCGGA GGAGATGCTCAC-3′, reverse: 5′-TACCCAAAGAATG GCCAAGT-3′; for human LGR-5, forward: 5′-GAGT TACGTCTTGCGGGAAAC-3′; reverse: 5′-TGGGT ACGTGTCTTAGCTG ATTA-3′ ; for actin, forward: 5′-CTGAGCGTGGCTACTCCTTC-3′, reverse: 5′-GCC ATCTCGTTCTCGAAGTC-3′. Relative fold changes in qPCR were determined by the ΔΔCt method.

### Immunohistochemistry assessment

All slides were assessed by one pathologist and one investigator independently and were blind to the cases. Firstly, the intensity of the Kindlin-1 immunostaining was scored as follows: 0, no color; 1, yellow; 2, brown; 3, dark brown. Then the number of Kindlin-1 positive cells was scored as follows: 1, ≤ 10% positive cells; 2, 11-50% positive cells; 3, 51-75% positive cells; 4, > 75%. These two score were multiply together. Those got 3 scores were defined as 1+; 4 as 2+; ≥5 as 3+, 1+ and 2+ were defined as positive and 3+ was defined as highly positive.

### Statistical analysis

Statistical analysis was performed with the SPSS for Windows, release 10. To test the significance of differences among groups of clinicopathological parameters, ordinal data were analyzed with the χ^2^ tests. The significance level was defined as *p*< 0.05. For Kindlin-1 and Ki-67 correlation evaluation, we count the positive cells of Kindlin-1 and Ki-67 in the adjacent places in serial sections. Bivariate correlations test was used to test the correlation of Kindlin-1 and Ki-67 expression. Other statistical tests were analyzed using GraphPad Prism 5. Data are presented as mean ± SEM and less frequently are presented as mean ± SD. Comparisons between two groups were made using 2-tailed Student's t test. Differences among more than two groups were compared using one-way ANOVA. P value less than 0.05 was considered statistically significant.
